# Case Fatality of Hospitalized Patients with COVID-19 Infection Suffering from Acute Respiratory Distress Syndrome in Germany

**DOI:** 10.3390/v14112515

**Published:** 2022-11-14

**Authors:** Ingo Sagoschen, Karsten Keller, Johannes Wild, Thomas Münzel, Lukas Hobohm

**Affiliations:** 1 Department of Cardiology, University Medical Center Mainz (Johannes Gutenberg-University Mainz), 55131 Mainz, Germany; 2 Center for Thrombosis and Hemostasis (CTH), University Medical Center Mainz (Johannes Gutenberg-University Mainz), 55131 Mainz, Germany; 3 Medical Clinic VII, Department of Sports Medicine, University Hospital Heidelberg, 69120 Heidelberg, Germany; 4 German Center for Cardiovascular Research (DZHK), Partner Site Rhine Main, Germany

**Keywords:** COVID-19, ARDS, case-fatality, trends, regional differences

## Abstract

**Aims:** Patients suffering from viral pneumonia caused by severe acute respiratory syndrome coronavirus 2 (SARS-CoV-2) infection are at risk of developing acute respiratory distress syndrome (ARDS). ARDS is a serious complication of COVID-19 that requires early recognition and comprehensive management. Little is known about the concomitant prevalence of both entities in Germany. Thus, we sought to analyze predictors and regional trends of case fatality in patients with COVID-19 and ARDS in Germany. **Methods:** We analyzed data on the characteristics, comorbidities and in-hospital outcomes for all hospitalized patients with COVID-19 and compared those with and without ARDS in Germany in 2020. **Results:** Overall, 176,137 hospitalized patients with confirmed COVID-19 were included in this analysis; among these, 11,594 (6.6%) suffered from ARDS. Most patients with ARDS were treated in hospitals in urban areas (*n* = 6485); proportion rate of mechanical ventilation was higher (45.9%) compared to those treated in hospitals of suburban (36.1%) or rural areas (32.0%). Proportion of ARDS grew exponentially with age until the sixth decade of life. Case-fatality rate was considerably higher in COVID-19 patients with ARDS compared to those without (48.3% vs. 15.8%; *p* < 0.001). Independent predictors of in-hospital case fatality with an OR > 3 were age ≥ 70 years, severe ARDS, severe liver disease, acute renal failure, stroke, dialysis treatment, shock and necessity of ECMO. **Conclusions:** The case fatality of COVID-19 patients with ARDS is dramatically high and shows relevant regional disparities. Our findings may help to draw more attention to predictors for in-hospital case fatality in patients hospitalized with COVID-19 and suffering from ARDS.

## 1. Take-Home Message

Severe pneumonia with consecutive acute respiratory distress syndrome requiring mechanical ventilation support occurs in 10–20% of hospitalized COVID-19 patients, with a high mortality rate. Our study provides new data about the in-hospital course of COVID-19 patients and ARDS. The prevalence of ARDS was 6.6%, with exponential increase with age and a dramatically high case-fatality rate of 48.3%. Our results could guide the attention on and increase the awareness for this life-threatening course of COVID-19 in this special population, with regard to health care coordination and ventilated intensive care unit bed availability.

## 2. Introduction

The novel coronavirus disease (COVID-19) quickly spread worldwide, starting with first cases from China at the end of 2019 [1,2]. In Germany, first cases of confirmed COVID-19 were reported in January 2020 [3]. Pneumonia caused by the SARS-CoV-2 virus is the most frequent severe respiratory complication, with concomitant fever and dyspnea, and can aggravate to acute respiratory failure due to acute lung injury [4]. Severe pneumonia with consecutive acute respiratory distress syndrome (ARDS) requiring mechanical ventilation support occurs in 10–20% of hospitalized COVID-19 patients [5]. In general, ARDS is a life-threatening disease entity with a high mortality rate (and an overall pooled mortality rate of 43%) [6,7]. ARDS is characterized by bilateral lung infiltrates and severe progressive hypoxemia in the absence of any evidence of cardiogenic pulmonary oedema [8,9]. After an acute phase of diffuse alveolar damage, interstitial oedema starts within seven days of the inciting event, followed by proliferation of fibroblast, which accelerates the transition to an organised stage [10]. ARDS definition and severity grading includes the paO2/FiO2 ratio, starting with values < 300. Since higher positive end-expiratory pressure (PEEP) may prevent end-expiratory alveolar collapse and therefore improve oxygenation, higher intra-pulmonary pressure may result in alveolar over-distension, followed by volume and atelectatic trauma, which is accompanied by increased inflammation and leads to a circulous vitiosus [11]. Besides these typical deviations in ARDS in general, acute lung injury and ARDS caused by SARS-CoV-2 seems to have different clinical presentations compared to classic ARDS. Several descriptions and explanations have been published since the beginning of the pandemic but no evidence based management changes have been established [12,13,14,15,16]. According to the latest publications, these disease-specific deviations might especially affect the early phase of the acute lung injury, ending in the well-known clinical manifestation comprising hypoxemia, low compliance and pulmonary fluid overload due to inflammation.

Since the beginning of the pandemic, worldwide deaths related to COVID-19 have surpassed 5 million deaths, including more than 100,000 deaths in Germany.COVID-19-patients with respiratory failure had an especially high case-fatality rate [17,18]. Thus, our aim was to provide detailed information about patient characteristics and outcomes of hospitalized patients with COVID-19 and ARDS in Germany in order to identify predictors of in-hospital case fatality for that critically ill patient group.

## 3. Materials and Methods

### 3.1. Source of Data and Ethics

The computed study analyses were performed on our behalf by the Research Data Center of the Federal Statistical Office and the Statistical Offices of the federal states in Wiesbaden, Germany (source: DRG Statistics 2020, own calculations). The Research Data Center provided us aggregated statistical results on the basis of SPSS codes (SPSS^®^ software, version 20.0, SPSS Inc., Chicago, IL, USA), which were sent by us to the Research Data Center. Since this study did not involve direct access to data of individual patients by the investigators, approval by an ethics committee and informed consent were not required, in accordance with German law.

### 3.2. Codes Regarding Diagnoses and Procedures

In Germany, diagnoses are coded according to the International Classification of Diseases and Related Health Problems, 10th Revision with German Modification (ICD-10-GM) and diagnostic, surgical or interventional procedures according to the German Procedure Classification (OPS, surgery and procedures codes [Operationen- und Prozedurenschlüssel]). All DRG (Diagnosis-Related Groups) diagnoses and OPS codes of hospitalized patients are gathered by the Federal Statistical Office of Germany. Therefore, we were able to identify all hospitalized patients with a confirmed COVID-19 diagnosis (ICD-code U07.1) and ARDS (ICD code J80) during the observational period between 1 January and 31 December 2020.

### 3.3. Parameters, Definitions and Study Outcomes

Analysed variables as comorbidities and clinical presentation comprised coronary artery disease [ICD-code I25], cancer [ICD-codes C00-C97], heart failure [ICD-code I50], atrial fibrillation/flutter [ICD-code I48], chronic obstructive pulmonary disease [ICD-code J44], arterial hypertension [ICD-code I10], chronic renal insufficiency [ICD-code N18.3, N18.4, N18.5, N18.83, N18.84, N18.9] and diabetes mellitus [ICD-code E10, E11, E12, E13, E14]. Outcomes of this study comprised death of any cause during the hospital stay (all-cause in-hospital death), major adverse events such as ischemic stroke [ICD-code I63] or clinically relevant bleeding events with the necessity of transfusion of erythrocyte concentrates [OPS code 8-800], intracerebral bleeding [ICD-code I61] or gastrointestinal bleeding [ICD-code I60]).

### 3.4. Statistical Methods

While continuous variables are presented as median and interquartile range (IQR), categorical variables were provided and reported as absolute numbers and corresponding percentages. Comparison of survivors versus non-survivors was performed using the Mann-Whitney-U test for continuous variables and the Fisher’s exact or chi-square test, as appropriate, for categorical variables. The total numbers, proportion, incidence, relative mortality rate and length of in-hospital stay for patients with COVID-19 and ARDS were calculated monthly, and linear regressions were used to assess trends over time. The results were presented as beta (β) and corresponding 95% confidence intervals (CI).

Univariate and multivariate logistic regression models were performed to investigate the impact of age, comorbidities and clinical conditions on mortality during hospitalization (in-hospital mortality). Results were presented as Odds Ratios (OR) and corresponding 95% CIs. Multivariate logistic regression models included the following parameters for adjustment: age, sex, cancer (ICD-codes C00-C97), coronary artery disease (ICD-code I25), heart failure (ICD-code I50), chronic obstructive pulmonary disease (COPD, ICD-code J44), essential arterial hypertension (ICD-code I10), diabetes mellitus (ICD-codes E10-E14) and chronic renal insufficiency (comprised diagnosis of chronic renal insufficiency stages 3 to 5 with glomerular filtration rate <60 mL/min/1.73 m^2^, ICD-code N18.3, N18.4, N18.5, N18.83, N18.84, N18.9) in order to demonstrate statistical independence of the aforementioned important parameters.

The software SPSS (SPSS^®^ software, version 20.0, SPSS Inc., Chicago, IL, USA) was used for statistical analysis. *p* values of <0.05 (two-sided) were considered to be statistically significant.

## 4. Results

### 4.1. Baseline Characteristics

**Figure 1:**
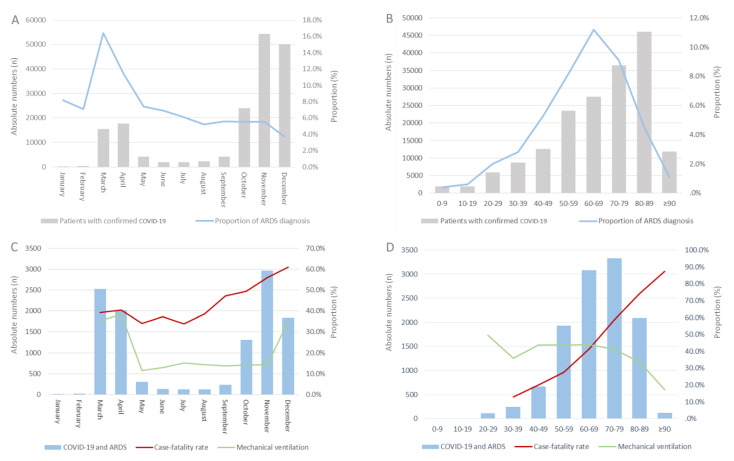
Proportion rate of ARDS diagnosis in patients with COVID-19 across months (**A**) and age decades (**B**). Case-fatality rate and proportion of mechanical ventilation in patients with COVID-19 and ARDS across months (**C**) and age decades (**D**) in Germany in 2020.

We analyzed data of 176,137 patients with confirmed SARS-CoV-2 infection hospitalized in Germany in 2020. Among these, 11,594 (6.6%) suffered from ARDS (Table 1). Although absolute numbers of COVID-19 cases increased, the proportion of ARDS decreased in 2020 (β −1.78 [95% CI −1.84 to −1.72]; *p* < 0.001) (Figure 1A). Regarding age, the proportion of ARDS grew exponentially until the sixth age decade, but when taking all age decades into account, this increase was not statistically significant (β 0.03 [95% CI −0.005 to 0.07]; *p* = 0.091) (Figure 1B).

The majority of patients with COVID-19-related ARDS were male (70.9%), had a median age of 69 and a median hospital length of stay (LOS) of 16 days (Table 1). Cardiovascular comorbidities were more frequently observed in patients with COVID-19 and ARDS than in COVID-19 patients without ARDS; coronary artery disease, heart failure, peripheral artery disease, atrial fibrillation/flutter and chronic obstructive pulmonary disease were more often present in COVID-19 patients with ARDS than in those without ARDS, whereas cancer and chronic renal insufficiency were more often coded in COVID-19 patients without ARDS (Table 1). Patients with COVID-19 and ARDS developed more often adverse events during hospitalization, such as deep vein thrombosis and/or thrombophlebitis, pulmonary embolism, acute kidney failure, severe liver disease or stroke (Table 1). The case-fatality rate was considerably higher in COVID-19 patients with ARDS than in patients without ARDS (48.3% vs. 15.8%; *p* < 0.001).

### 4.2. Trends of In-Hospital Case Fatality in Patients with COVID-19 and ARDS

**Figure 2:**
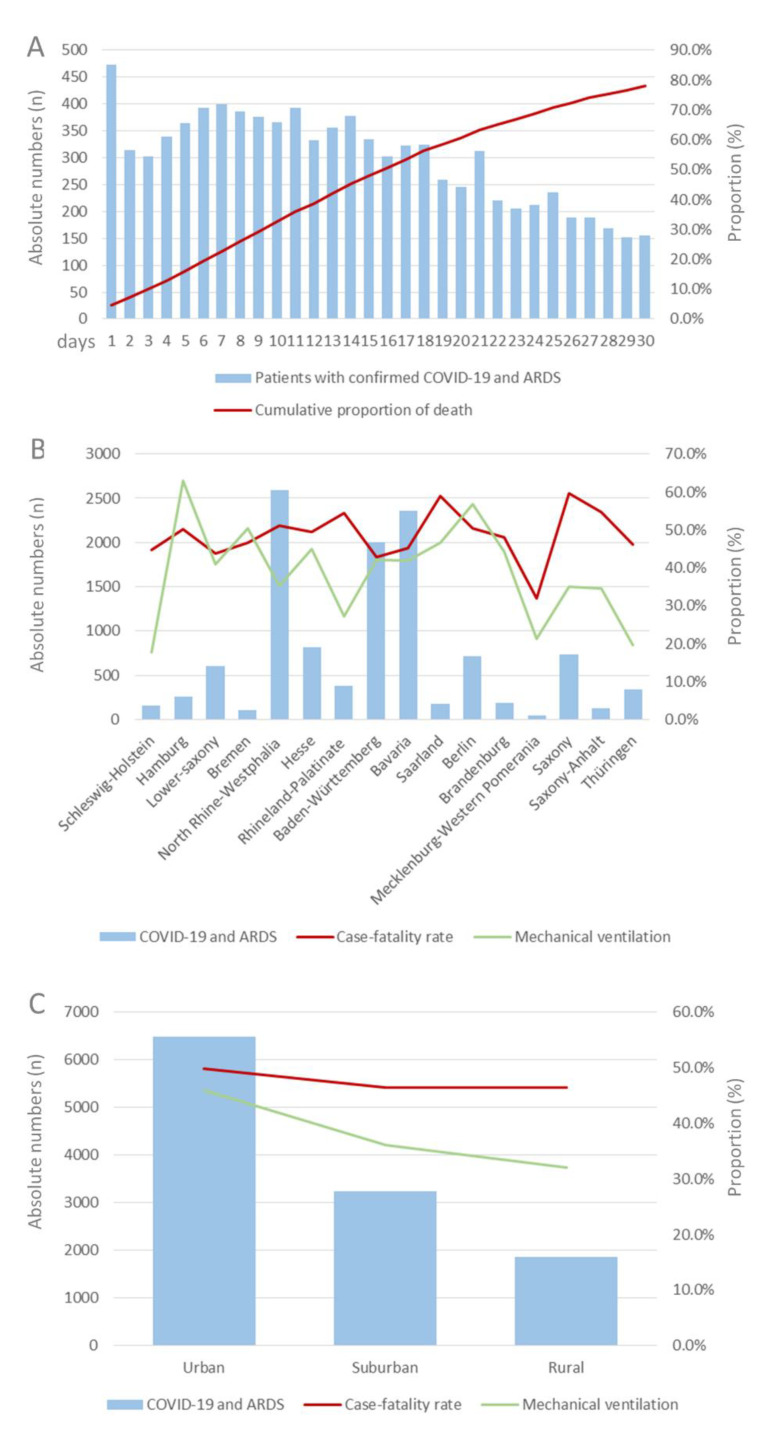
Cumulative proportion of deaths during in-hospital stay in patients with COVID-19 and ARDS (**A**), as well as trends regarding absolute numbers, case-fatality rates and mechanical ventilation, stratified for regions (**B**) and urban–rural (**C**) differences in Germany in 2020.

Overall, 5604 (48.3%) patients with COVID-19 and ARDS died during the in-hospital stay. Both case fatality and mechanical ventilation were highest in months with a high number of COVID-19 cases (case fatality: β 1.24 [95% CI 1.10 to 1.37]; *p* < 0.001 and mechanical ventilation: β 0.44 [95% CI 0.30 to 0.58]; *p* < 0.001) (Figure 1C). A substantial age-dependent increase of case fatality could be observed (β 0.97 [95% CI 0.92 to 1.01]; *p* < 0.001), whereas mechanical ventilation was decreasingly used with increasing age (β −0.18 [95% CI −0.23 to −0.13]; *p* < 0.001) (Figure 1D). Almost half (45.2%) of the deaths of COVID-19 patients with ARDS occurred during the first 14 days after hospital admission (Figure 2A).

### 4.3. Predictors of In-Hospital Case Fatality and Its Regional Disparities

Non-survivors were older and had a considerably aggravated comorbidity profile, including higher rates of coronary artery disease, cancer, heart failure, atrial fibrillation/flutter, chronic renal insufficiency, diabetes mellitus and peripheral artery disease. As expected, serious events such as bleeding with necessity of transfusion of erythrocyte concentrates, ischaemic stroke, pulmonary embolism or myocardial infarction were more prevalent in non-survivors compared to survivors (Table 2). Independent predictors of in-hospital mortality in the multivariate logistic regression model with an OR > 3 were: age ≥ 70 years, severe ARDS, necessity of ECMO, severe liver disease, acute renal failure, stroke, dialysis, shock and cardio-pulmonary resuscitation (Table 3). 

Most COVID-19 patients with ARDS were treated in hospitals in urban areas (*n* = 6485) showing a high case-fatality rate (49.8%) but also a concomitant high proportion rate of mechanical ventilation (45.9%), compared to hospitals in suburban (36.1%) or rural areas (32.0%) (Figure 2C). While the lowest case-fatality rate of COVID-19 patients with ARDS was observed in the federal state of Mecklenburg-Western Pomerania (31.9%) as opposed to the federal city-states Berlin (50.4%) and Hamburg (50.2%), the highest use of MV in COVID-19 patients with ARDS was reported for Berlin (56.8%) and Hamburg (50.2%) as well (Figure 2B).

## 5. Discussion

The aim of the present study was to examine patient characteristics, regional differences and, in particular, outcomes of hospitalized COVID-19 patients with ARDS in German hospitals in 2020. During this period, the wild-type of the SARS-CoV-2 Virus was predominant in Germany. with prevalence of the alpha variant rising in December 2020. Since the beginning of the pandemic, worldwide deaths related to COVID-19 have constantly increased, and in this context, loss of life expectancy helps to compare how different countries have experienced, and been impacted by, the COVID-19 pandemic [19]. For example, US life expectancy decreased by an historic 1.87-year reduction in 2020 compared to 2019. In Germany, a moderate decrease in life expectancy was observed, while three countries (New Zealand, Norway and South Korea) gained life expectancy between 2019 and 2020 [19]. Since the majority of COVID-19 cases passed as an asymptomatic or mild infection, the manifestation of a respiratory infection with hypoxaemic failure is known as a strong predictor of in-hospital case fatality [18]. Already in the pre-COVID era, ARDS was known for its often fatal prognosis, with a reported in-hospital mortality of 34.9% for mild cases to 46.1% for those with severe ARDS [20].

A recent meta-analysis reported a comparable pooled mortality of 39% (95% CI: 23–56%), also among COVID-19 patients with ARDS across all countries [21]. As expected, the observed case-fatality rate differs worldwide. The highest case-fatality rates among patients with COVID-19 and ARDS were found in China (69% [95% CI: 67–72%]), as opposed to Spain and France, which had considerably lower case-fatality rates (40% [95 CI: 39–41%] and 19% [95% CI: 17–20%]). The pooled mortality estimate in Europe was 34% (95% CI: 20–50%). The high rate of case fatality in China might be explained by the initial outbreak of the pandemic in China, whereas Europe may benefit from a better understanding of the COVID-19 when the outbreak shifted after some weeks from China to Europe. In the present analysis, the case-fatality rate of Germany (48.1%) was in-between the rates of other countries. This detected case-fatality rate in the present analysis of the nationwide inpatient sample of Germany is much higher than estimated in a previous publication, which is highly explainable due to the fact that only 24 COVID-19 patients with ARDS until April 2020 were included in those previous estimations [22].

As demonstrated in a recent meta-analysis, the case-fatality rate is substantially higher among older patients, with more than 70% deceased patients aged over 60with mechanical ventilation [23]. Accordingly, our study also demonstrated a considerable and strong association between age and case fatality, especially in patients aged ≥70, who had a 3.61-fold (95% CI: 3.41–4.01) risk for an adverse outcome. In line with previous data, our cohort also showed a higher prevalence of ARDS in male COVID-19 patients. It is already known that patients with ARDS are predominantly male, with or without COVID-19 [24]. Risk factors and differences were observed with regard to hormones, which can influence inflammation and immunological function between sexes [25].

The management of patients with COVID-19 has been adapted over the course of the COVID-19 pandemic, and the use of extracorporeal membrane oxygenation (ECMO) has increased [26]. Patients with the necessity of ECMO had a cumulative incidence of in-hospital mortality, 90 days after initiating ECMO, of 37.4% (95% CI: 34.4–40.4) [27]. In the present analysis, one-tenth of the patients with COVID-19 and ARDS had to undergo ECMO implantation. Those patients were at 7.99-fold (95% CI: 6.89–9.28) increased risk for an adverse outcome.

Although Germany has a highly developed health care system, our present study demonstrated large regional differences regarding the hospitalization and in-hospital case fatality in Germany. As expected, most patients with ARDS were treated in hospitals in urban areas (*n* = 6485) with a high case-fatality rate (49.8%), but also with a concomitant high proportion rate of mechanical ventilation (45.9%). Those proportion rates reflect the so-called cloverleaf principle (German: “Kleeblatt Struktur”), which supports and sustains overburdened regions and hospitals by transferring patients to regions with free hospital capacities. With respect to resource availability and allocation, patients with critical illness were mostly transferred from rural to urban hospitals for therapy escalation. Thus, there is an expected higher in-hospital case-fatality rate in urban hospitals. Several authors point out that “COVID-19 ARDS” has significant pathophysiological differences compared to “typical ARDS” and is considered to be more complex and diverse. Previous literature postulated that patients with COVID-19 pneumonia often presented with unique “silent” hypoxemia, which can lead to a severe stage of ARDS, based on the Berlin criteria, even if the patients were not ventilated or in intensive care units [28]. 

The main results of the study can be summarized as follows:

(i). In COVID-19 patients, the proportion of ARDS was 6.6%, with exponential increase with increasing age until the sixth age decade and highest prevalence in winter 2020.
(ii). Case-fatality rate was considerable higher (48.3%) in COVID-19 patients with ARDS compared to those without ARDS (15.8%).
(iii). Most patients with ARDS were treated in hospitals in urban areas (*n* = 6485) with a high case-fatality rate (49.8%) but also with a concomitant high proportion of patients requiring mechanical ventilation (45.9%).
(iv). Independent predictors of in-hospital death in the multivariate logistic regression model with an OR > 3 were age ≥ 70 years, severe ARDS, necessity of ECMO, severe liver disease, acute renal failure, stroke, dialysis, shock and cardio-pulmonary resuscitation.

Although this study includes data collected on a national level for around 180,000 hospitalized patients with COVID-19, we recognize that this study analysis has some limitations. First, as our results are based on administrative and retrospective data, we cannot exclude misclassification or inconsistencies. Notably, COVID-19 pneumonia is not yet defined with separate criteria, and thus a separate ICD-code is not present so far. This can lead to miscoding or over-reporting of ARDS cases. Second, this analysis of the German nationwide inpatient sample was not prespecified; therefore, our findings can only be considered to be hypothesis-generating. Third, patients with confirmed COVID-19 who died out of hospital or were diagnosed post mortem after coding were not included in the German nationwide inpatient sample. Fourth, the German nationwide inpatient sample does not report long-term outcomes following discharge from hospital. Fifth, changes in treatment recommendations at prophylactic or even therapeutic doses, as well anti-inflammatory regimens, were not considered in this analysis due to missing codes in the nationwide sample.

Our findings demonstrated a dramatically high case-fatality rate in patients with COVID-19 when suffering from ARDS. Our results could guide the attention on, and increase the awareness of, this life-threatening course of COVID-19 in this special population, with regard to healthcare coordination and ventilated intensive care unit bed availability.

## Figures and Tables

**Table 1:** Characteristics, medical history, presentation and adverse in-hospital events of the 176,137 hospitalized patients with confirmed COVID-19 in Germany in 2020, stratified for the presence of Acute Respiratory Distress Syndrome (ARDS).

Parameters	COVID-19 without ARDS (*n* = 164,543; 93.4%)	COVID-19 with ARDS (*n* = 11,594; 6.6%)	*p*-Value
**Age**	72.0 (55.0/82.0)	69.0 (59.0/78.0)	**<0.001**
**Age ≥ 70 years**	88,785 (54.0%)	5544 (47.8%)	**<0.001**
**Female sex**	80,570 (49.0%)	3379 (29.1%)	**<0.001**
**In-hospital stay (days)**	7.0 (3.0/13.0)	16.0 (8.0/29.0)	**<0.001**
**VTE risk factors**			
**Obesity**	7919 (4.8%)	1464 (12.6%)	**<0.001**
**Diabetes mellitus**	41,042 (24.9%)	4190 (36.1%)	**<0.001**
**Essential arterial hypertension**	76,456 (46.8%)	6024 (52.0%)	**<0.001**
**Comorbidities**			
**Coronary artery disease**	23,589 (14.3%)	1985 (17.1%)	**<0.001**
**Heart failure**	24,513 (14.9%)	2606 (22.5%)	**<0.001**
**Peripheral artery disease**	5554 (3.2%)	416 (3.6%)	**0.016**
**Atrial fibrillation/flutter**	30,882 (18.8%)	3278 (28.3%)	**<0.001**
**Chronic obstructive pulmonary disease**	11,131 (6.8%)	1023 (8.8%)	**<0.001**
**Chronic renal insufficiency (glomerular filtration rate < 60 mL/min/1.73 m^2^)**	25,716 (15.6%)	1656 (14.3%)	**<0.001**
**Cancer**	8473 (5.1%)	528 (4.6%)	**0.005**
**Charlson comorbidity index**	4.0 (2.0/6.0)	5 (3.0/7.0)	**<0.001**
**Respiratory manifestations of COVID-19**			
**Pneumonia**	95,699 (58.2%)	11,214 (96.7%)	**<0.001**
**ARDS mild**	0 (0%)	529 (4.6%)	**<0.001**
**ARDS moderate**	0 (0%)	2764 (23.8%)	**<0.001**
**ARDS severe**	0 (0%)	7912 (68.2%)	**<0.001**
**Treatment**			
**Intensive care unit**	17,484 (10.6%)	9569 (82.5%)	**<0.001**
**Mechanical ventilation**	7395 (4.5%)	4747 (40.9%)	**<0.001**
**Extracorporeal membrane oxygenation (ECMO)**	147 (0.1%)	1307 (11.3%)	**<0.001**
**Dialysis**	2936 (1.8%)	2639 (22.8%)	**<0.001**
**Adverse events during hospitalization**			
**Transfusion of blood constituents**	9526 (5.8%)	4348 (37.5%)	**<0.001**
**Deep vein thrombosis**	1467 (0.9%)	316 (2.7%)	**<0.001**
**Pulmonary embolism**	2602 (1.6%)	760 (6.6%)	**<0.001**
**Myocardial infarction**	2389 (1.5%)	355 (3.1%)	**<0.001**
**Acute kidney failure**	16,307 (9.9%)	5768 (49.7%)	**<0.001**
**Severe liver disease**	2891 (1.8%)	1248 (10.8%)	**<0.001**
**Myocarditis**	161 (0.1%)	65 (0.6%)	**<0.001**
**Stroke (ischaemic or haemorrhagic)**	2788 (1.7%)	408 (3.5%)	**<0.001**
**Shock**	3009 (1.8%)	3377 (29.1%)	**<0.001**
**Cardio-pulmonary resuscitation**	1796 (1.1%)	1063 (9.2%)	**<0.001**
**Case fatality**	26,003 (15.8%)	5604 (48.3%)	**<0.001**

**Table 2:** Characteristics, medical history, presentation and adverse in-hospital events of the 11,594 hospitalized patients with confirmed COVID-19 and concomitant acute respiratory distress syndrome (ARDS) in Germany in 2020, stratified for the presence of in-hospital death.

Parameters	Survivors (*n* = 5990; 51.7%)	Non-Survivors (*n* = 5604; 48.3%)	*p*-Value
**Age**	63.0 (55.0/72.0)	74.0 (65.0/81.0)	**0.001**
**Age ≥ 70 years**	1930 (32.2%)	3614 (64.5%)	**<0.001**
**Female sex**	1727 (28.8%)	1652 (29.5%)	**0.449**
**In-hospital stay (days)**	21.0 (10.0/36.0)	13.0 (7.0/21.0)	**0.001**
**VTE risk factors**			
**Obesity**	835 (13.9%)	629 (11.2%)	**<0.001**
**Diabetes mellitus**	2042 (34.1%)	2148 (38.3%)	**<0.001**
**Essential arterial hypertension**	3180 (53.1%)	2844 (50.7%)	**0.012**
**Heart failure**	987 (16.5%)	1619 (28.9%)	**<0.001**
**Comorbidities**			
**Coronary artery disease**	774 (12.9%)	1211 (21.6%)	**<0.001**
**Peripheral artery disease**	138 (2.3%)	278 (5.0%)	**<0.001**
**Atrial fibrillation/flutter**	1290 (21.5%)	1988 (35.5%)	**<0.001**
**Chronic obstructive pulmonary disease**	406 (6.8%)	617 (11.0%)	**<0.001**
**Chronic renal insufficiency (glomerular filtration rate < 60 mL/min/1.73 m^2^)**	571 (9.5%)	1085 (19.4%)	**<0.001**
**Cancer**	193 (3.2%)	335 (6.0%)	**<0.001**
**Charlson comorbidity index**	4.0 (2.0/6.0)	6.0 (5.0/8.0)	**<0.001**
**Respiratory manifestations of COVID-19**			
**ARDS mild**	414 (6.9%)	115 (2.1%)	**<0.001**
**ARDS moderate**	1874 (31.3%)	890 (15.9%)	**0.001**
**ARDS severe**	3489 (58.2%)	4423 (78.9%)	**<0.001**
**Treatment**			
**Intensive care unit**	4793 (80.0%)	4776 (85.2%)	**<0.001**
**Mechanical ventilation**	2616 (43.7%)	2131 (38.0%)	**<0.001**
**Extracorporeal membrane oxygenation (ECMO)**	391 (6.5%)	916 (16.3%)	**<0.001**
**Dialysis**	749 (12.5%)	1890 (33.7%)	**<0.001**
**Adverse events during hospitalization**			
**Transfusion of blood constituents**	1809 (30.2%)	2539 (45.3%)	**<0.001**
**Myocardial infarction**	120 (2.0%)	235 (4.2%)	**<0.001**
**Deep vein thrombosis**	205 (3.4%)	111 (2.0%)	**<0.001**
**Pulmonary embolism**	354 (5.9%)	406 (7.2%)	**0.004**
**Acute kidney failure**	2087 (34.8%)	3681 (65.7%)	**<0.001**
**Severe liver disease**	284 (4.7%)	964 (17.2%)	**<0.001**
**Stroke (ischaemic or haemorrhagic)**	125 (2.1%)	283 (5.0%)	**<0.001**
**Shock**	1003 (16.7%)	2374 (42.4%)	**<0.001**
**Cardio-pulmonary resuscitation**	248 (4.1%)	815 (14.5%)	**<0.001**

**Table 3:** Impact of baseline characteristics, comorbidities, clinical presentation and complications on in-hospital case fatality.

	All Patients with COVID and ARDS (*n* = 11,594; 5604 Patients Died In-Hospital [48.3%])			
	Univariate		Multivariate (Adjusted for Age, Sex, Cancer, Coronary Artery Disease, Heart Failure, COPD, Arterial Hypertension, Renal Insufficiency and Diabetes Mellitus)	
Parameters	OR (95% CI)	*p*-Value	OR (95% CI)	*p*-Value
**Age**	1.10 (1.08–1.11)	0.001	1.07 (1.06–1.07)	**0.001**
**Age ≥70 years**	3.82 (3.54–4.13)	<0.001	3.61 (3.41–4.01)	**<0.001**
**Sex (female)**	1.03 (0.95–1.12)	0.443	0.89 (0.82–0.98)	**0.018**
**Obesity**	0.78 (0.70–0.87)	<0.001	1.10 (0.97–1.25)	**0.121**
**Comorbidities**				
**Coronary artery disease**	1.86 (1.68–2.05)	<0.001	1.09 (0.98–1.22)	**0.129**
**Cancer**	1.91 (1.59–2.29)	<0.001	1.86 (1.53–2.26)	**<0.001**
**Heart failure**	2.06 (1.88–2.25)	<0.001	1.43 (1.29–1.58)	**<0.001**
**Atrial fibrillation/flutter**	2.01 (1.85–2.17)	<0.001	1.22 (1.11–1.33)	**<0.001**
**COPD**	1.70 (1.49–1.94)	<0.001	1.25 (1.09–1.44)	**0.002**
**Essential arterial hypertension**	0.91 (0.85–0.98)	0.012	0.69 (0.63–0.75)	**<0.001**
**Chronic renal insufficiency**	2.279 (2.04–2.54)	<0.001	1.36 (1.21–1.54)	**<0.001**
**Diabetes mellitus**	1.20 (1.11–1.29)	<0.001	1.01 (0.93–1.11)	**0.722**
**pAVK**	2.21 (1.79–2.72)	<0.001	1.31 (1.05–1.64)	**0.017**
**Charlson index**	1.43 (1.41–1.45)	<0.001	1.37 (1.35–1.41)	**<0.001**
ARDS subgroups *				
**ARDS mild**	0.28 (0.23–0.35)	<0.001	0.21 (0.16–0.26)	**0.001**
**ARDS moderate**	0.41 (0.38–0.45)	<0.001	0.34 (0.30–0.37)	**0.001**
**ARDS severe**	2.69 (2.47–2.92)	<0.001	3.56 (3.25–3.91)	**0.001**
**Treatment modalities**				
**Intensive Care Unit**	1.44 (1.31–1.58)	<0.001	1.85 (1.66–2.06)	**<0.001**
**Mechanical Ventilation**	0.79 (0.73–0.85)	<0.001	0.85 (0.78–0.92)	**0.001**
**ECMO**	2.79 (2.47–3.17)	<0.001	7.99 (6.89–9.28)	**<0.001**
**Serious adverse events through hospitalization**				
**Ischaemic stroke**	2.49 (2.02–3.01)	<0.001	3.07 (2.44–3.86)	**0.001**
**Transfusion of erythrocytes**	1.91 (1.77–2.06)	<0.001	2.24 (2.06–2.44)	**<0.001**
**Severe liver disease**	4.17 (3.64–4.79)	<0.001	5.67 (4.87–6.61)	**<0.001**
**Acute renal failure**	3.58 (3.31–3.86)	<0.001	3.21 (3.15–3.43)	**<0.001**
**Dialysis**	3.56 (3.24–3.91)	<0.001	4.47 (4.02–4.98)	**<0.001**
**Deep vein thrombosis**	0.57 (0.45–0.72)	<0.001	0.69 (0.54–0.89)	**0.001**
**Pulmonary embolism**	1.24 (1.07–1.44)	0.004	1.58 (1.34–1.85)	**0.001**
**Myocardial infarction**	2.14 (1.71–2.67)	<0.001	1.46 (1.15–1.86)	**0.002**
**Shock**	3.65 (3.35–3.98)	<0.001	4.15 (3.77–4.57)	**<0.001**
**Cardio-pulmonary resuscitation**	3.94 (3.40–4.56)	<0.001	4.66 (3.97–5.47)	**<0.001**

Abbreviations: COPD = Chronic obstructive pulmonary disease; OR = Odds ratio; CI = Confidence interval. Values in bold indicate that the difference is statistically significant, at least in the multivariate regression model (*p* < 0.05). * In 3.4% of all cases, the ARDS subgroups were not defined.

## Data Availability

All code used in this study is publicly available online. The data used in this study are sensitive due to individual patient-level data and will not be made publicly available. The data is available at the Federal Statistical Office of Germany (Statistisches Bundesamt, DEStatis) (source: RDC of the Federal Statistical Office and the Statistical Offices of the federal states, DRG Statistics 2020, and own calculations).

## Abbreviations

ARDS	Acute respiratory distress syndrome
CI	Confidence interval
COPD	Chronic obstructive pulmonary disease
CPR	Cardio-pulmonary resuscitation
DRG	Diagnosis-Related Groups
ICD	International Classification of Disease
IQR	Interquartile range
NIS	Nationwide inpatient sample
NYHA	New York Heart Association
OPS	Surgery and interventional procedure keys (Operationen- und Prozedurenschlüssel)
OR	Odds ratio
SARS-CoV-2	Severe acute respiratory syndrome coronavirus 2
